# Parasitism, Emergence, and Development of *Spalangia endius* (Hymenoptera: Pteromalidae) in Pupae of Different Ages of *Bactrocera cucurbitae* (Diptera: Tephritidae)

**DOI:** 10.1093/jisesa/ieu180

**Published:** 2015-02-19

**Authors:** Liang-De Tang, Xun-Cong Ji, Yun Han, Bu-Li Fu, Kui Liu

**Affiliations:** ^1^Environment and Plant Protection Institute, Chinese Academy of Tropical Agricultural Sciences, Haikou 571101, China; ^3^Hainan Academy of Agricultural Sciences, Institute of Agreoenvironment and Plant Protection, Hainan Key Laboratory for Control of Plant Diseases and Insect Pests, Haikou 571100, China; ^4^Engineering Research Center of Biological Control, Ministry of Education, College of Natural Resource and Environment, South China Agricultural University, Guangzhou 510642, China

**Keywords:** parasitoid, melon fly, pupal age, host suitability, biological control

## Abstract

The wasp* Spalangia endius* Walker (Hymenoptera: Pteromalidae) is a major parasitoid of the pupae of fruit flies, which are a common agricultural pest. An understanding of this intricate host–parasitoid interaction could provide basic information necessary for the sustainable integrated biological control of fruit flies. In this study, we investigated the effect of *S. endius* on different-aged pupae of the melon fly *Bactrocera cucurbitae* Coquillett by using choice and nonchoice tests under laboratory conditions. We showed that *S. endius* females oviposited, and their progeny successfully developed, in different-aged pupae of* B. cucurbitae* regardless of the method of exposure. There was an oviposition preference for 3–5-d-old pupa. The highest mean percentage parasitism occurred on 4- and 5-d-old hosts, followed by 2- and 3-d-old hosts. The average development time for both males and females was significantly longer in 6–7-d-old hosts than in the younger host stages. Adult females that developed from younger host pupae (2–5-d old) were significantly heavier than those from older host pupae (6–7-d old), and they also lived longer. The sex ratio (proportion of females) of the parasite progeny decreased with an increase in host age. Host mortality also decreased gradually as the pupal age increased. The differences in development time, body weight, and longevity between females and males were significant. These results suggest that *S. endius* is a good candidate for the biological control of *B. cucurbitae*.

Insect hosts can provide important resources for the growth and development of parasitoid offspring; therefore, host quality is a crucial factor for parasitoid fitness (Godfray 1993, Harvey et al*.* 1994). The nutritional quality of the host can influence the parasitoid offspring in terms of their body size, fecundity, longevity, foraging efficiency, developmental rate, sex ratio, and survival of immature stages (Harvey and Vet 1997, Harvey 2001). However, the quality of the host can depend on its species, size, developmental stage, and whether it had been previously parasitized. Therefore, female parasitoids have evolved the ability to recognize different host factors, such as host size and age, and whether the host has been previously parasitized. Female parasitoids use olfactory, tactile, gustatory, and visual cues to locate a suitable host species and then to distinguish between different developmental stages or sizes of the same host (Godfray 1993, Nofemela and Kfir 2008). A female parasitoid completes the host-selection process by using her ovipositor. When she comes into contact with a host, the female inserts her ovipositor into the host to further evaluate its suitability and food quality, and then decides whether to oviposit (Godfray 1993).

Studies have suggested that parasitoid oviposition decisions are based on optimal foraging theory and optimal oviposition theory (Charnov 1976, Stephens and Krebs 1987, Charnov and Stephens 1988). The optimal foraging theory, also called the oviposition preference offspring performance hypothesis, postulated that, when provided with several ages of hosts that differ in quality, a female parasitoid should preferentially parasitize the highest quality host (Wang and Liu 2002). The optimal oviposition theory suggests that mothers preferentially lay female eggs in high-quality hosts and male eggs in low-quality hosts (King et al*.* 1993, King 2000).

There are differences in host quality associated with the increasing age of the host, as a result of the histolysis of internal tissues, the formation of adult internal organs, and the sclerotization of appendages; therefore, although pupal size does not change with age, older host pupae might contain fewer resources for parasitoid development compared with younger pupae of a comparative size (Chapman 1998). Although some parasitoid species prefer to oviposit on medium-aged host pupae (Pfannenstiel et al*.* 1996), most species prefer to oviposit on younger host pupae because these generally offer higher quality nutrition for subsequent development (King 2000, Husni et al*.* 2001). Additionally, parasitoid wasps also have the ability to determine the sex of their offspring at oviposition, which enables females to adapt their sex allocation in relation to host quality (the haplodiploid sex determination system; Charnov and Stephens 1988, King 2000).

The melon fruit fly, *Bactrocera cucurbitae* Coquillett (Diptera: Tephritidae) is a worldwide quarantine pest. It damages >100 species of vegetables and fruits and, in China, can have four to six generations per year (Li et al*.* 2008, Ou et al*.* 2008). Given that the fruits and vegetables are harvested over relatively short-time intervals and are then marketed for human consumption almost immediately, it is difficult to use chemical insecticides as a major method of controlling the melon fly. Therefore, several methods (e.g., bagging of fruits, protein baits, cue-lure traps, host plant resistance, biological control, and soft insecticides) have been used to suppress this pest. Of these, biological control could have an important role in suppressing the fruit fly population and is perhaps environmentally friendly than some other methods.

*Spalangia endius* Walker (Hymenoptera: Pteromalidae) is a well-known parasitoid of certain fly species (Rueda and Axtell 1985) and is used to control these dipterous pests. During our study, *S. endius* was frequently found in association with not only *B. cucurbitae* but also *Bactrocera* *dorsalis* in orchard and vegetable fields in southern China (unpublished). Therefore, *S. endius* could be a potential biological control agent against fruit flies in China. However, *S. endius* strains from different areas might have important different biological consequences. Moreover, there is a lack of information on the relation between *S. endius* and *B. cucurbitae*, particularly in terms of what determines host choice. Thus, this study examined the suitability of, and preference for, different host stages of *B. cucurbitae* for *S. endius*. These results could not only maximize the use of *S. endius* as a biological control agent in integrated pest management programs but also determine the optimum time for its release in the field.

## Materials and Methods

### 

#### Experimental Materials

A colony of *S. endius* was established from parasitized *B. dorsalis* pupae obtained from orchards and continuously reared on *B. dorsalis* in insect rearing room. Hatched larvae of *B. dorsalis* were kept in groups until pupation in crisper (4 liters). Larvae were fed with a banana-based artificial diet that was renewed every 3 d until pupation. Emerged adult parasitoids (50–100 pairs) were placed inside oviposition cages (25 by 25 by 30 cm) and supplied with cotton wool saturated with 15% honey and water. Parasitization was accomplished by placing mated 2-d-old adult female *S. endius* in oviposition cages containing 500 *B. dorsalis* individuals for 48 h. The females used had not previously oviposited.

The *B. cucurbitae* host pupae were collected from vegetable fields and reared on a pumpkin-based artificial diet (Wang et al. 2012). To obtain synchronized cohorts of different stages of host pupae for the study, newly pupated *B. cucurbitae* were collected daily for 6 d from laboratory colonies and kept separately according to their cohort in 2-, 3-, 4-, 5-, 6-, and 7-d-old groups; thus, six different pupal-stage groups (2-, 3-, 4-, 5-, 6-, and 7-d-old host pupae) were obtained, which were separated and used for the study. One-day-old *B. cucurbitae* pupae were not used because of high mortality rates in preliminary experiments.

The cultures and experiments were conducted in a climate-controlled room, at 26 ± 1°C, 65 ± 5% relative humidity (RH), and a 14:10 (L:D) h photoperiod.

#### Choice Test

Six pupal stages of *B. cucurbitae* were exposed simultaneously to six female parasitoids in a single experimental cage (15 by 15 by 20 cm) for 24 h. In total, 180 host pupae (30 of each stage) were placed randomly in the cage. At the end of the exposure period, host pupae from each treatment were placed in 100-ml plastic cups covered with a little humid sand and kept at 25–27°C until wasps had emerged. The opening of the plastic cup was covered with a fine nylon mesh (120 meshes).

Twenty-five replicates for each treatment were performed, and 4,500 host pupae were used in the tests. Host pupae were observed on daily basis. For each host pupal stage, the numbers of hosts parasitized, unparasitized, and dead, the number of parasitoid adults that exited the host, but failed to reach adulthood, and the number of parasitoids that died inside the pupae were recorded. Pupae that died were dissected and observed under a stereomicroscope to distinguish between parasitized and no parasitized hosts.

#### No-Choice Test

The procedure for the no-choice test was similar to that of the choice tests. Thirty host pupae from each of the 2-, 3-, 4-, 5-, 6-, and 7-d-old groups were placed separately in a 500-ml glass bottle, and a mated 2-d-old adult female parasitoid was introduced at random. After 24 h, the parasitoid was removed, and the pupae were placed at 25–27°C in 100-ml plastic cups covered with a little humid sand until wasps had emerged. The opening of the plastic cup was again covered with a fine nylon mesh (120 meshes).

Thirty-one replicates for each treatment were performed, and 5,580 host pupae were used in the tests. Host pupae were observed on daily basis. For each pupal stage, the number of hosts parasitized or unparasitized, the number of emerged parasitoids that failed to pupate, and the number of parasitoids that died inside the pupae were recorded.

#### Suitability Test by Observation

Host suitability was evaluated by analyzing the influence of host age on different biological parameters of *S. endius*. To test the effect of host pupal age on the development of *S. endius* progeny, 2-d-old female parasitoids were exposed to host pupae as described above for the choice test. Each experimental cage (15 by 15 by 20 cm) contained 100 host pupae of a particular stage and eight female parasitoids. Trials with each stage (2–7-d-old groups) were replicated 10 times. After exposure, the experiment was conducted following the same procedure as described above for the choice test.

For each host pupal stage, the duration of the immature stage, body weight, sex ratio, and adult longevity were determined.

#### Data Analysis

The number of host pupae parasitized, as estimated by observing parasitoid adult emergence and by dissection, was used to calculate the relative parasitism index (*P*_i_) of each of the six age classes in the series of choice tests (equation 1):

(1) $$P_{i}=\frac{R_{i}}{\sum_{i=1}^{m}Ri}$$

where *P_i_* is the relative parasitism index of the stage group *i*, *R_i_* is the number of host pupae parasitized in stage group *i*, and *m* is the total number of stage groups tested in a test (six stage groups altogether in this case).

The percentage of parasitism of *S. endius* was estimated based on equation 2:

(2) $$\text{Parasitism percentage}\left(\%\right)=\left(N_{\text{p}}/N_{\text{t}}\right)*\text{1}00$$

where, *N*_p_ is the number of parasitized pupae, and *N*_t_ is the total number of host pupae that were parasitized in one treatment (=30 here).

Host mortality in the treatment, including death as a result of being unsuccessfully parasitized and stung, and so on, was corrected by the death data collected in the control for each host age group, according to Abbott (1925). The corrected mortality (%) (*M*_corrected_) was calculated according to equation 3:

(3) $$\text{Mortality}\left(\%\right)=\left[\left(M_{\text{treatment}}–\text{Parasitism}\left(\%\right)–M_{\text{control}}\right)/\left(\text{1}00–M_{\text{control}}\right)\right]*\text{1}00$$

One-way analysis of variance was used to compare the *B. cucurbitae* pupal-stage groups in terms of the number of hosts parasitized, development time, offspring longevity, emergence rate, host mortality, body weight, and sex ratio of emergent parasitoids. All proportional data were normalized through arcsine transformation for all statistical analyses, the level of significance was set at 5%, and the analyses were performed using SPSS 16.0.

## Results

### 

#### Host Preference Study

To test the preference for different-aged pupae of *B. cucurbitae*, six pupal stage groups were offered simultaneously or separately to female wasps. Female *S. endius* parasitized pupae of all ages and their offspring were able to develop to adulthood in pupae of all ages in both the choice and no-choice tests. However, the *S. endius* females showed a preference for 3–5-d-old pupae in both the choice and no-choice tests (Tables 1 and 2). The percentage parasitism in the 3–5-d-old pupal groups was significantly higher compared with the 2-, 6-, and 7-d-old pupal groups in either test (Tables 1 and 2).

**Table 1.** Effect of host pupal age on percentage of host pupae parasitized, parasitoid emergence, and host mortality in the no-choice test

Pupae age (d)	Parasitism percentage (%)	Emergence percentage (%)	Mortality (%)
Second	22.8 ± 2.01b	99.0 ± 059a	14.5 ± 1.14a
Third	35.1 ± 1.88a	98.9 ± 0.70a	12.8 ± 1.33ab
Fourth	32.9 ± 1.99a	99.3 ± 0.38a	11.0 ± 0.79bc
Fifth	31.3 ± 1.48a	99.3 ± 0.40a	10.1 ± 0.82bc
Sixth	19.0 ± 1.65b	99.3 ± 0.46a	8.7 ± 0.89c
Seventh	8.4 ± 1.32c	99.2 ± 1.29a	5.5 ± 0.44d

*N* = 30 host pupae in each age group. Means followed by same letter in columns (Duncan’s test) do not differ statistically (*P* > 0.05).

**Table 2.** Effect of host pupal age on percentage of host pupae parasitized, mean parasitism index, parasitoid emergence, and host mortality in the choice test

Pupae age (d)	Parasitism (%)	Mean parasitism index (*P_i_*)	Emergence (%)	Mortality (%)
Second	15.2 ± 0.64b	0.13 ± 0.006b	98.4 ± 1.13a	10.6 ± 1.53a
Third	23.5 ± 2.01a	0.23 ± 0.008a	98.9 ± 0.62a	9.6 ± 0.91ab
Fourth	25.1 ± 1.49a	0.22 ± 0.009a	99.5 ± 0.50a	9.5 ± 2.29 ab
Fifth	24.3 ± 1.89a	0.22 ± 0.008a	99.6 ± 0.40a	8.0 ± 1.39 ab
Sixth	15.2 ± 1.11b	0.14 ± 0.009b	99.6 ± 0.36a	7.8 ± 1.71 ab
Seventh	7.7 ± 0.69c	0.06 ± 0.006c	100.0 ± 0.00a	5.7 ± 1.02b

*N* = 180 host pupae across groups, with 30 pupae in each age group. Means followed by same letter in columns (Duncan’s test) do not differ statistically (*P* > 0.05).

Both in the choice and no-choice tests, there was no significant difference in emergence percentage of parasitoids from parasitized hosts among the different pupal age groups, and all percentages were >98%. The mortality of the hosts gradually decreased with increasing host pupal age, with peak mortality occurring in the 2-d-old group (14.5% for no-choice test and 10.6% for choice test); minimum mortality occurred in the 7-d-old pupal group (5.5% for no-choice test and 5.7% for choice test) (Tables 1 and 2).

#### Host Suitability Study

The development time, body weight, and longevity of female parasitoids were significantly affected by the particular pupal stage parasitized. Mean development time of *S. endius* was significantly longer for females from 6- and 7-d-old pupae compared with other host ages; body weight and longevity for female *S. endius* were significantly higher for 2–5-d-old pupal groups compared with those in other pupal ages, although the differences between the four groups were not significant.

The pupal age of the host parasitized had no effect on mean body weight, development time, or longevity of the male offspring (Table 3), being around 0.19 mg, 21 d, and 9.7 d, respectively, regardless of the pupal age of the host in which they were reared. The development time of female parasitoids was 5–10% longer than males, whereas their longevity was also longer by approximately 10–20% (Table 3). Furthermore, female parasitoids had nearly double the body weight of males even when reared on the same-aged host. Progeny resulted from eggs laid across the pupal-age groups showed a significant female bias, although the proportion of females varied according to host pupal age, with the highest occurring in the 2–5-d-old pupae, followed by those emerged 1 d earlier than lighter progeny reared on 6–7-d-old pupae (Table 3).

**Table 3.** Development time, body weight, male progeny ratio, and adult longevity of *S. endius* reared on host pupae of different ages

Pupae age (d)	Developmental time (d)		Body weight (mg)		Sex ratio (% male)	Adult longevity (d)	
	Female	Male	Female	Male		Female (*n* = 30)	Male (*n* = 30)
Second	21.6 ± 0.19b	20.6 ± 0.23*a	0.38 ± 0.011a	0.20 ± 0.006*a	35.0 ± 0.64 a	11.7 ± 0.49a	10.1 ± 0.26*a
	(*n* = 106)	(*n* = 68)	(*n* = 72)	(*n* = 66)			
Third	21.5 ± 0.19b	20.5 ± 0.17*a	0.39 ± 0.009a	0.20 ± 0.004*a	36.1 ± 1.12a	11.6 ± 0.46a	10.0 ± 0.32*a
	(*n* = 105)	(*n* = 73)	(*n* = 73)	(*n* = 70)			
Fourth	21.6 ± 0.18b	20.6 ± 0.26*a	0.37 ± 0.008a	0.20 ± 0.007*a	37.3 ± 0.59a	11.9 ± 0.42a	9.8 ± 0.40*a
	(*n* = 106)	(*n* = 61)	(*n* = 61)	(*n* = 48)			
Fifth	21.5 ± 0.25b	20.6 ± 0.31a*	0.37 ± 0.007a	0.19 ± 0.008*a	37.7 ± 1.56a	11.8 ± 0.43a	10.0 ± 0.41*a
	(*n* = 101)	(*n* = 51)	(*n* = 66)	(*n* = 52)			
Sixth	22.5 ± 0.25a	20.7 ± 0.26*a	0.33 ± 0.011b	0.18 ± 0.010*a	45.6 ± 2.32b	10.3 ± 0.38b	9.3 ± 0.32*a
	(*n* = 69)	(*n* = 42)	(*n* = 41)	(*n* = 36)			
Seventh	22.5 ± 0.27a	20.8 ± 0.31*a	0.3 ± 0.011b	0.19 ± 0.007*a	54.5 ± 1.34c	10.0 ± 0.23b	9.2 ± 0.43*a
	(*n* = 56)	(*n* = 36)	(*n* = 31)	(*n* = 34)			

Means followed by same letter in columns (Duncan’s test) do not differ statistically (*P* > 0.05).

*A significant difference between females and males (Student’s *t* test; *P* < 0.05).

## Discussion

Different hosts, different host pupal age, or different host instars can have different nutrition qualities (Harvey et al*.* 2000, Harvey and Strand 2002). The offspring of hymenopteran parasitoids rely on their host for all their nutritional needs, and, therefore, most parasitoids have the ability to determine host quality during oviposition (Husni et al. 2001, Quimio and Walter 2001, Wang and Liu 2002, Roriz et al. 2006, Karut 2007, He et al. 2011). Most studies have shown that wasps prefer to oviposit on younger host pupae, which provide higher-quality nutrition (Takagi 1985; Ueno 1997; King 1998, 2000; Husni et al*.* 2001; Wang and Liu 2002). However, some studies have shown that parasitoids prefer medium-aged host pupae for development (Pfannenstiel et al*.* 1996). Our results showed that the S. endius– *B. cucurbitae* association follows the latter in that intermediate-aged host pupae were parasitized more often than older or younger pupae. This relationship appears to be in close agreement to that of Kitthawee et al. (2004) for S. endius–*Bactrocera correcta* association. Differences in the number of pupae parasitized among different host ages could result from several factors, such as more difficulty exiting from older pupae, nutrient deficiency of older pupae, and fewer drills (Chapman 1998, Wang and Liu 2002, Beckage and Gelman 2004, Henry et al*.* 2005). In contrast, the results of our study showed that there was high mortality of parasitized 2-d-old pupae, such that it would be better for females to avoid ovipositing in these younger pupae. Our study showed a decrease in mortality rate with an increase in age of pupa, suggesting that young pupae are more susceptible to the injuries caused at oviposition, including sting or venom injection or both. In contrast, parasitism did not seem to induce mortality in older pupa (Colinet et al*.* 2005). The lower number of pupae parasitized observed in older hosts could reflect defensive behavioral responses of these hosts.

Some studies have reported that the development time of parasitoid wasps varied with host age, such as *Mythimna separata* (Walker) (Husni et al*.* 2001), *Diadromus collaris* (Wang and Liu 2002), *Nasonia vitripennis* (Wylie 1964), and *Dinarmus basalis* (Islam 1994). Moreover, some wasp species developed to a larger size when they were reared on younger hosts (Ueno 1997, King 1998, Husni et al*.* 2001). In this study, the female offspring produced in younger pupae (2–5-d-old pupae) were significantly heavier in terms of their body weight compared with older hosts (6–7-d-old pupae). Adult progeny of *S. endius* that were heavier emerged earlier than lighter progeny when reared on 2–7-d-old host pupae, and, similarly, heavier progeny lived longer compared with lighter progeny. Therefore, we can assume that host age has an effect on the nutrition available for the development of the parasitoid progeny, resulting in older host pupae being less suitable for the rearing of *S. endius*.

Some factors are known to influence the sex ratio of parasitoid progeny, such as parental sex ratio (Mohamed and Coppel 1986), host size (Barbosa and Frongillo 1979), and host age (Barbosa and Frongillo 1979, Barbosa et al*.* 2009). Ueno (1997, 1999) found that parasitoids (*Itoplectis naranyae* and *Pimpla nipponica*) could change the sex ratio of their offspring in response to host age. In our study, we found that the highest proportion of males occurred in 6–7-d-old hosts (Table 3). In contrast, Husni et al*.* (2001) reported that the age of the host *M. separata* had no effect on the progeny sex ratio of the wasp *Brachymeria lasus*.

In China, the area under protected culture and greenhou*s*e vegetable planting has expanded rapidly in recent years, and *B. cucurbitae* has become a dominant pest in these areas. Moreover, to our knowledge, there are no published reports on the successful use of natural enemies against *B. cucurbitae*, and the parasitization of *B. cucurbitae* by native parasitoid species is thought to be insignificant. Although *S. endius* can be a hyperparasite, it would still be possible to release this species in the fields to act as a biological control agent. However, there is a need to evaluate the parasitization potential of *S. endius* before its exploitation as an effective biocontrol agent for the management of *B. cucurbitae*.

In conclusion, we demonstrated that *S. endius* can develop successfully in 1–7-d-old host pupae but preferred to parasitize 3–5-d-old host pupae. The parasitism percentage was higher in 3–5-d-old hosts in both the choice and no-choice test. Female adult progeny from young (2–5-d old) hosts were heavier, had a shorter development time, and longer longevity compared with those from older hosts (6–7-d old). These results suggest that *S. endius* is a suitable candidate for the biological control of *B. cucurbitae*. Our results will be useful in designing mass-rearing protocols, and further biological control studies of *B. cucurbitae* under greenhouse and field conditions.
